# Exploring emerging IoT technologies in smart health research: a knowledge graph analysis

**DOI:** 10.1186/s12911-020-01278-9

**Published:** 2020-10-08

**Authors:** Xuejie Yang, Xiaoyu Wang, Xingguo Li, Dongxiao Gu, Changyong Liang, Kang Li, Gongrang Zhang, Jinhong Zhong

**Affiliations:** 1 The School of Management, Hefei University of Technology, 193 Tunxi Road, Hefei, 230009 Anhui China; 2 The 1st Affiliated Hospital, Anhui University of Traditional Chinese Medicine, 117 Meishan Road, Hefei, 230031 Anhui China; 3 Key Laboratory of Process Optimization and Intelligent Decision-making of Ministry of Education, Hefei, 230009 Anhui China

**Keywords:** Internet of things, Disease, Health, Bibliometrics, Visual analysis

## Abstract

**Background:**

At present, Internet of Things technology has been widely used in various fields, and smart health is also one of its important application areas.

**Methods:**

We use the core collection of Web of Science as a data source, using tools such as CiteSpace and bibliometric methods to visually analyze 9561 articles published in the field of smart health research based on the Internet of things (IoT) in 2003–2019, including time distribution, spatial distribution, and literature co-citation analysis and keyword analysis.

**Results:**

The field of smart health research based on IoT has developed rapidly since 2014, but has not yet formed a stable network of authors and institutions. In addition, the knowledge base in this field has been initially formed, and most of the published literatures are multi-theme research.

**Conclusions:**

This study discusses the research status, research hotspots and future development trends in this field, and provides important knowledge support for subsequent research.

## Background

The Internet of Things (IoT) is an important representative of the new generation of information technology. It is the result of rapid development in the field of wireless communications in recent years, and it is a network that extends on the Internet [1]. It can connect various information sensing devices (such as Radio Frequency Identification, infrared sensors, laser scanners, etc.) to the Internet to realize the “Internet of Everything” [2]. At present, IoT has been widely used in various fields, such as smart city, smart home, intelligent logistics, intelligent transportation, etc. Among them, smart health is also one of its important application areas. There are countless people who lose their lives every year due to various diseases or health problems. In terms of chronic diseases, the number of people dying from chronic diseases accounts for 60% of the total number of deaths worldwide. People are paying more and more attention to health issues [3]. Therefore, the use of IoT technology to solve health problems has become one of the research hotspots in the field of smart health.

IoT is connecting physical world with virtual world of Internet. Physical world includes household appliances (such as air purifiers, thermostats, etc.), automobiles, industrial machinery, construction, medical equipment, and human body [4]. Applying IoT technologies to healthcare will help improve the quality of people life, the level of chronic disease management, danger warning and life-saving interventions. There are lots potential applications of healthcare IoT: (1) *Health monitoring*. Today’s wearable devices can detect basic activities of human body, analyze human behavior, and measure health status. Smart wearable devices (such as smart watch) can reduce patient anxiety and reduce waste of resources [5]. This is very different from other sensitive devices for health monitoring in conventional hospitals. (2) *Health information support for patients*. You can remind patients to take medicine on time through some IoT devices, in clinical. Networking devices such as electrocardiogram, blood oxygen, and blood pressure can improve the continuous measurement, monitoring, and support structure of patients and caregivers, thereby improving clinical outcomes [6, 7]. (3) *Service improvement*. IoT can help connect cars to network systems. If a car has an accident, the system can identify the severity of the accident and help traffic administration department and healthcare emergency center via sending the accident location and direction. This will help the injured people obtain timely assistance [8]. (4) *The collection of information resource for big data analytics*. The health IoT can generate massive amounts of health big data. The analysis, mining, and use of health big data can further promote and enhance the development of health IoT [9].

Since 2003, scholars from all over the world have gradually invested in research in the field of smart health research based on the IoT. In response, some scholars have designed smart wearable systems to solve health problems. Li et al. [10] established a model of the acceptance of smart wearable system by the elderly, and pointed out the factors influencing their use of smart wearable technology, including self-reported health status. Akbulut et al. [11] designed a smart wearable system that monitors cardiovascular disease, which provides continuous medical monitoring. Fraise et al. [12] proposed a multi-agent system (MAS) that uses smart wearable and mobile technology to care for patients in elderly care facilities. In recent years, the development of technology has made smart watches and smart bracelets popular. Previously, Lu et al. [13] reviewed the application of smart watches in the field of medical health. Through comparative experiments, Hataji et al. [14] showed that combined treatment could improve the daily physical performance of patients with chronic obstructive pulmonary disease (COPD) under the encouragement of smart watches. Wile et al. [15] used smart watch devices to distinguish between orthostatic recurrent tremor and primary tremor of Parkinson’s disease. Grym et al. [16] pointed out that smart wristbands are a viable continuous monitoring tool during pregnancy. Smart home is also an important application of IoT technology in the field of smart health. Dawadi et al. [17] demonstrated the feasibility of using smart home sensor data and learning-based data analysis to predict clinical scores. Pham et al. [18] proposed a cloud-based smart home environment (CoSHE) for home healthcare. Ghasemi et al. [19] proposed a smart home medical system that can diagnose environmental events and health risks quickly and in a timely manner. Alberdi et al. [20] ‘s experiments show that all mobile, cognitive, and depressive symptoms can be predicted by activity-aware smart home data. In addition, research on disease and health issues through IoT technology is the focus of research in this field. Zhang et al. [21] proposed a medical data fusion algorithm based on IoT for the particularity of medical IoT data. Hossain et al. [22] proposed an industrial Internet of Things (IIoT) health monitoring framework that supports cloud computing. Farahani et al. [23] introduced the overall architecture of the fog-driven IoT e-health ecosystem and discussed the applicability and challenges of the IoT in the field of healthcare.

At present, there are many researches on the application of smart wearables, smart watches, smart bracelets, smart homes and IoT technologies to the field of smart health. However, there is no research to objectively review and visualize all the literature in this field. In order to analyze the development status and future trends of the intelligent health research field based on the IoT systematically, comprehensively, and objectively, this study uses bibliometric methods to visualize the analysis from time distribution, spatial distribution, literature co-citation and keywords based on 9561 literature data in this field from 2003 to 2019. This research provides panoramic knowledge support for researchers in related fields to understand the research status, future trends and hotspots in the field of smart health research based on IoT.

## Methods

### Data sources

The data source for this study was Web of Science (WoS), which selected four core databases of its core collections, including Science Citation Index Expanded, Conference Proceedings Citation Index-Science, and so on. WoS is an important database for obtaining global academic information. It contains more than 13,000 authoritative, high-impact academic journals from around the world, covering the fields of natural sciences, engineering technology, biomedicine, social sciences, arts and humanities. WoS includes references cited in the paper, with a unique citation index, users can use an article, a patent number, a conference document, a journal or a book as a search term to retrieve their citations and easily trace the origin and history of a research document, or track its latest progress. Although the WoS database cannot include all the literature published in this field, it has some representativeness. We invited 5 experts in the field of health IoT to finalize the database and search strategy through Delphi method. The search strategy we used is as follows: TS = (“#1” AND “#2”), Where “#1” is TS = (“internet of things” OR “smart watch*” OR “smart wristband*” OR “smart home*” OR “wearable device*” OR “wearable technolog*” OR “wearable sensor*”), indicating the search term related to the IoT; “#2” indicates TS = (“diseas*” OR “health*“OR “hospital*”), which indicates a search term related to health. In order to ensure that the retrieved documents are related to the retrieval subject, we organized a panel of evaluation consisting of eight Ph.D. candidates in our research area. After excluding irrelevant articles, we finally got 9561 records. (The search time was August 2020).

### Research methods and tools

This paper mainly adopts the method of bibliometrics. Bibliometrics refers to the quantitative analysis and management of literature information by mathematical and statistical methods, and then discusses its structure, characteristics and laws [24]. This study mainly uses HistCite, CiteSpace and MS Excel to visually analyze the relevant literature in the field of smart health research based on IoT. Because HistCite’s statistical function is relatively powerful, it is mainly used to collect relevant data in this paper, and then use Excel software to draw the chart [25]. CiteSpace is a visualization tool for bibliometrics that focuses on finding key points in the development of a field [26]. Therefore, this article mainly uses CiteSpace to visualize the authors, institutions, literature co-citation and keywords of the IoT-based smart health research field.

## Results

### Time distribution map

**Fig. 1:**
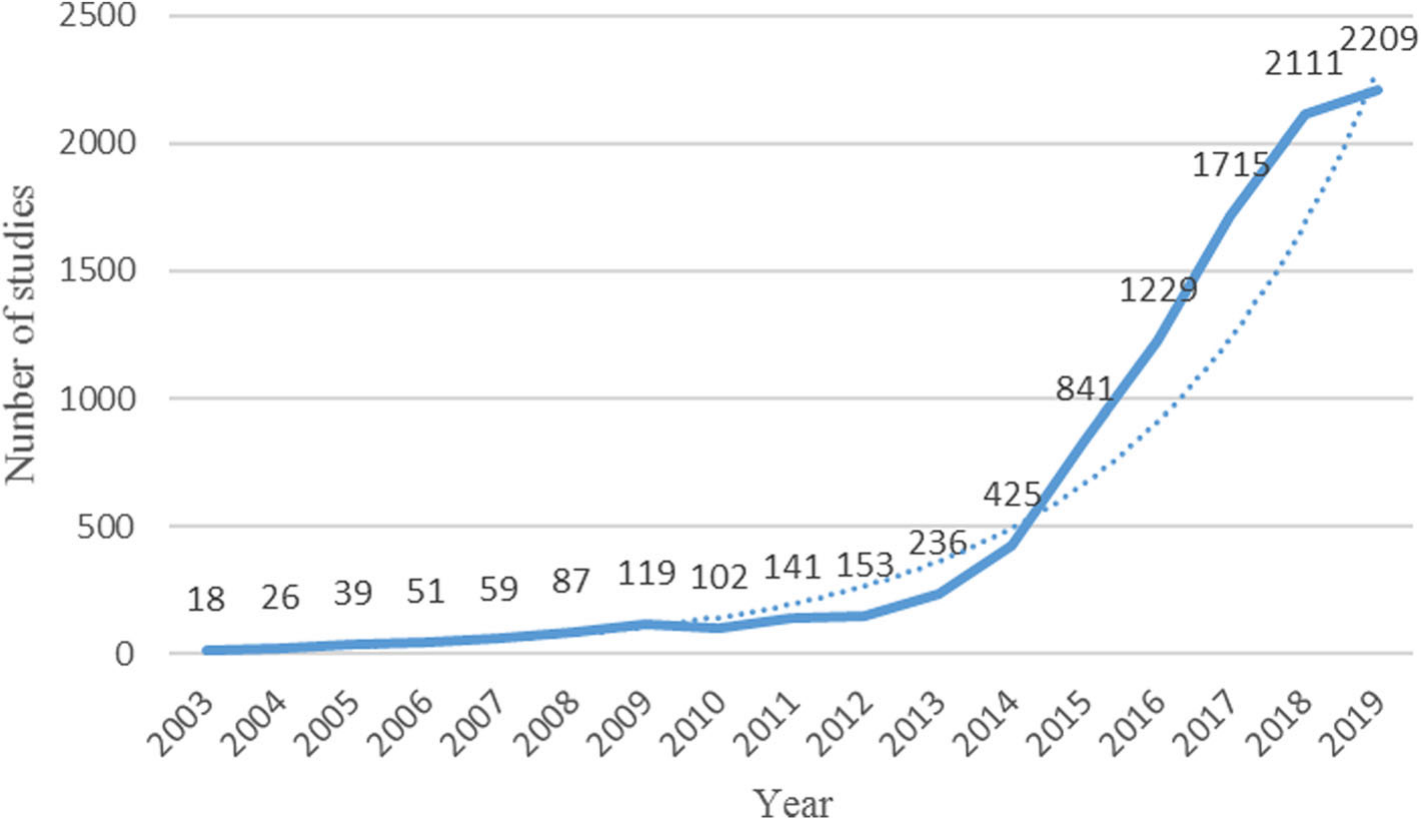
Annual number of published articles

In order to understand the output of research results in the field of smart health research based on IoT, HistCite was used to statistically analyze the number of scientific literatures in the years from 2003 to 2019, and the trend of annual papers was obtained, as shown in Fig. 1. As can be seen from the figure, from 2003 to 2019, the annual capacity curve presents an overall growth trend, among which the curve of annual capacity from 2003 to 2014 is relatively flat, and the annual capacity basically conforms to the exponential growth trend, or even below the exponential trend line. In 2014–2019, the annual capacity curve has grown very rapidly, almost showing a linear upward trend, much higher than the index trend line, with a growth rate of 97.88% in 2014–2015. This figure suggests that research in this field will continue to increase in the future and that it will remain a hotspot for future research.

**Fig. 2:**
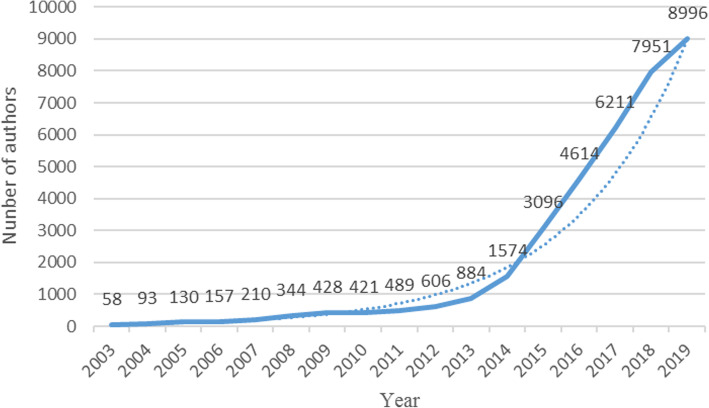
Annual number of authors input

Then, we explored the input of researchers in the field of smart health research based on IoT. The same use of HistCite to statistically analyze the number of scientific research participants in the years from 2003 to 2019, and get the trend of the annual author input, as shown in Fig. 2. By comparing Figs. 1 and 2, we can clearly find that their variation trend is roughly the same, in years with a high growth rate of annual capacity, the growth rate of author input is also high. It is also easy to understand that, generally speaking, the annual number of articles and the annual amount of authors input is directly proportional to the relationship.

**Fig. 3:**
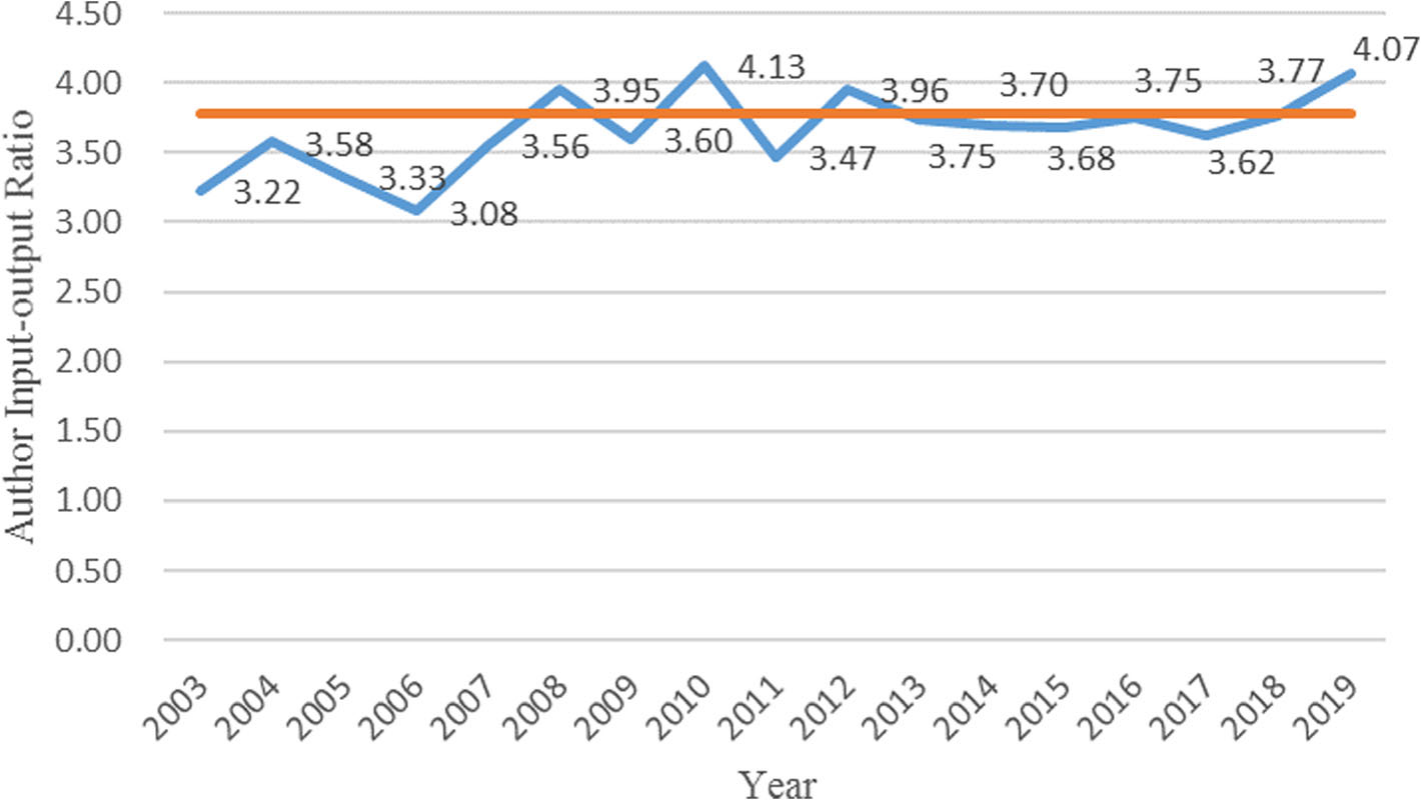
Annual author input-output ratio

Finally, the input-output ratio of scientific researchers in the field of smart health research based on IoT was understood. We calculated the number of participants in a single paper from 2003 to 2019, and obtained the change trend of the ratio of participants in a single paper, as shown in Fig. 3. The straight line parallel to the abscissa in the figure is the average number of participants in a single document over the years, which is 3.79. Overall, the annual input-output ratio fluctuated significantly, especially during 2003–2011. This was because the number of documents in previous years was small and the researchers were not fixed. Then the ratio is roughly stable. In the 17 years, only the authors in 2010 and 2019 had an input-output ratio of more than 4. The small number of participants in a single document indicates that in this research field, the contribution rate of individual authors is high, the cooperation between authors is less, the number of authors is not saturated, and there is still much room for improvement.

### Space distribution map

#### Author distribution

**Fig. 4:**
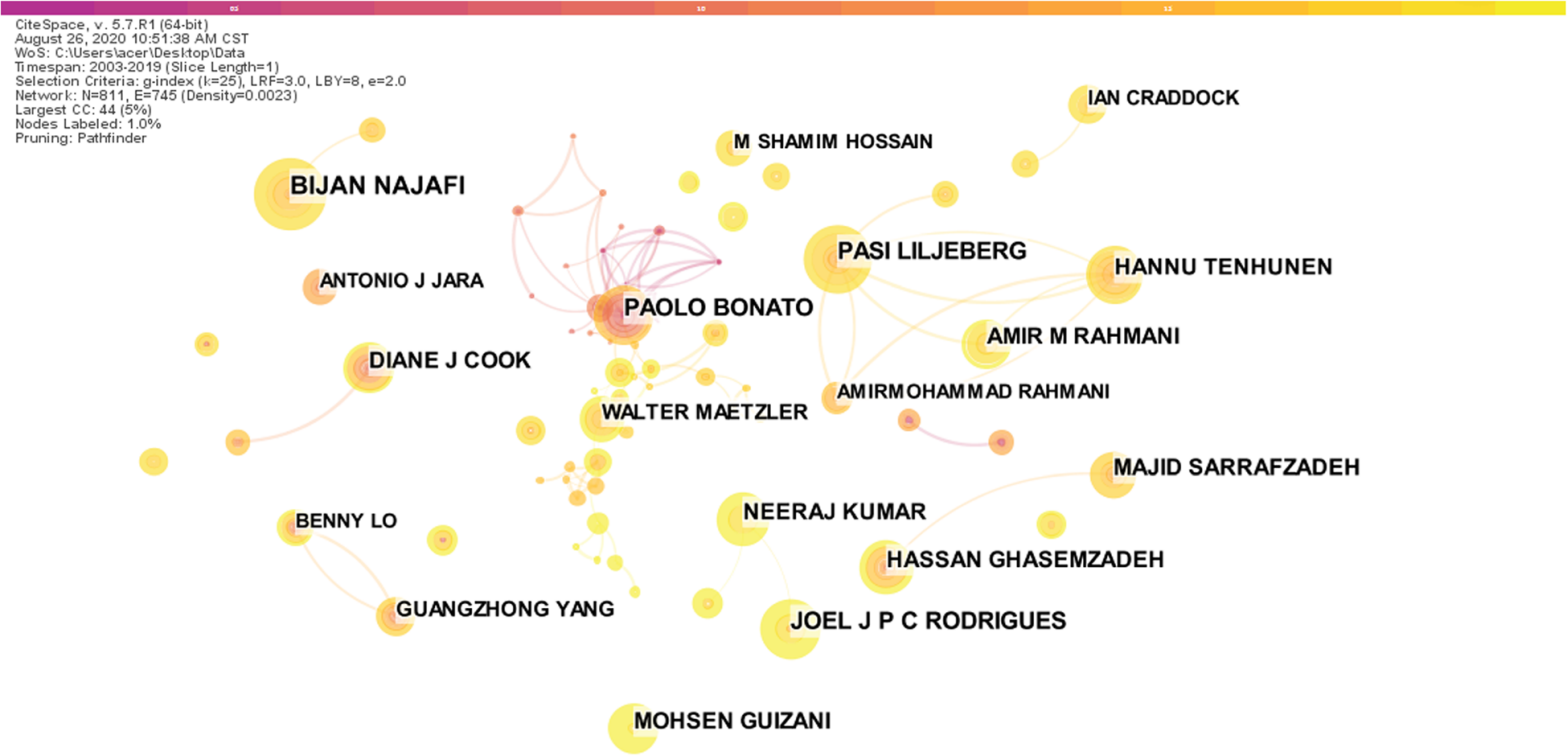
Author collaboration network

In order to analyze the author’s cooperative network, we import the preprocessed data into CiteSpace to generate the author cooperation network diagram, as shown in Fig. 4. The figure shows the author’s name and the relationship between the authors with 10 or more articles. The most published is Bonato and Rahmani, which has published 35 articles. In the figure, the size of the node is proportional to the number of articles issued by the author, the thickness of the connection between the nodes is proportional to the number of cooperation between the authors, and different colors indicate the year of cooperation between different authors. As can be seen in the upper left corner of Fig. 4, the number of network nodes is 811, the number of connections between nodes is 745, and the density of the network is only 0.0023.

Table 1 specifies the authors of the top 10 articles and their related information. In the HistCite software system, the citation frequency is divided into LCS and LGS, where LCS (local citation score) refers to the citation frequency of the document in the database of the field, and GCS (global citation score) refers to the frequency of citation in the Web of Science database [27]. It can be seen from Table 1 that Bonato and Rahmani have the largest number of publications, and the total quotation has reached 2024 and 908 respectively. The average single paper has been cited more than 15 times, indicating that his papers are not only issued more, but also have higher recognition. The sparseness of network density indicates that the cooperation between authors is not close enough, the field of smart health research based on IoT has not yet formed a stable core author group. And if the authors strengthen cooperation, they can collide with new sparks, promote research and innovation, and make the field flourish.

**Table 1:** The top 10 authors and their number of published articles

Author	Number of published articles	LCS	GCS
Bonato P	35	291	2024
Rahmani AM	35	260	908
Cook DJ	31	249	1284
Najafi B	30	73	442
Liljeberg P	28	192	674
Rodrigues JJPC	28	49	361
Tenhunen H	24	156	531
Kumar N	24	43	395
Ghasemzadeh H	23	98	517
Guizani M	21	31	417

#### Institutional distribution

**Fig. 5:**
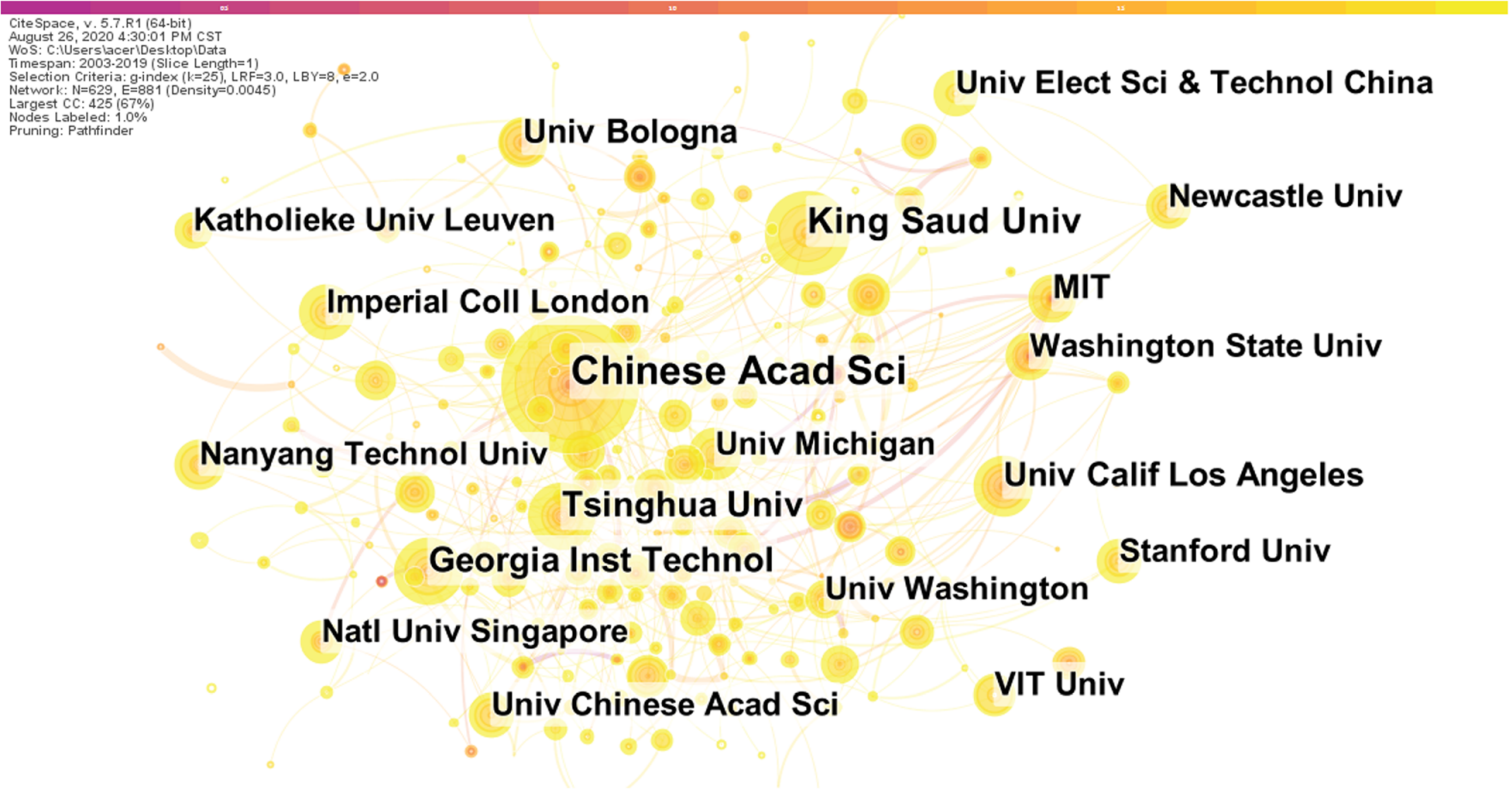
Institution collaboration network

Then we import the pre-processed data into CiteSpace to analyze the organization that publishes the scientific literature, and generate the organization cooperation network diagram, as shown in Fig. 5. Table 2 shows the top 10 organizations and related information. The organization with the largest number of publications is Chinese Academy of Sciences, which has published 134 scientific papers, 52 more than the second-ranked King Saud University. In addition, there are nearly 200 organizations that have published at least 10 articles, indicating that the research on the field of smart health research based on IoT has received extensive attention from various authoritative academic institutions in the world, showing a hundred schools of contention and a hundred flowers.

**Table 2:** The top 10 institutions in number of published articles

Institution	Number of published articles	LCS	GCS
Chinese Academy of Sciences	134	562	6399
King Saud University	82	287	1815
Georgia Institute of Technology	68	74	1818
Tsinghua University	65	133	1848
University of California, Los Angeles	64	130	1285
Massachusetts Institute of Technology	59	224	2242
University of Bologna	55	119	860
Imperial College London	53	70	878
Washington State University	51	326	1714
University of Michigan	51	61	894

According to Table 2, Chinese Academy of Sciences not only has the largest number of publications, but its LCS and GCS are also much higher than the second-ranked King Saud University. Although Georgia Institute of Technology is ranked third in the number of publications, its LCS is not high. The average number of citations in the field is only 1.09, indicating that the published articles are not highly recognized by peers. On the contrary, although the numbers of published literatures are not large by Massachusetts Institute of Technology and Washington State University, their average total number of citations have reached 38 and 33 respectively. At the same time, Chinese Academy of Sciences’ LCS and GCS data ranks first among all institutions. These explain that the quality of the literature published by these institutions is very high and is widely supported and highly recognized by researchers and peers.

Research cooperation between institutions is an important way to enhance the research strength of the organization as a whole, to achieve complementary scientific research resources and to share knowledge, and to reflect one of the important indicators of research status in a certain field [28]. In Fig. 5, the size of the node is proportional to the number of documents sent by the organization, the thickness of the connection between the nodes is proportional to the number of cooperative researches between the organizations, and different colors indicate the year of collaborative research between the institutions. The number of network nodes is 629, the number of connections between nodes is 881, and the density of the network is 0.0045. It can be seen that in the field of smart health research based on IoT, there is not enough cooperation between institutions, and the relationship between them is not close enough, and a stable and mature institutional cooperation relationship has not yet been formed. Institutions should strengthen cooperation and exchanges, give full play to their respective advantages, and make full use of academic resources to promote innovation in research results and promote the vigorous development of the entire field.

#### Journal distribution

Finally, we analyzed the journals in the field of smart health research based on IoT. We use HistCite to collect statistics on journals in the field, and Table 3 lists the top 10 journals and related information. Table 3 shows that the journal with the largest number of documents is *Sensors*, and its LCS and GCS data are also among the best. Interestingly, four of the top 10 journals are from the IEEE Publishing Group: *IEEE Access*, *IEEE Internet of Things Journal*, *IEEE Sensors Journal* and *IEEE Journal of Biomedical and Health Informatics.* The average single article of *IEEE Journal of Biomedical and Health Informatics* has been cited as high as 31.7, which is enough to see the journal’s influence in the field of smart health research based on IoT. Conversely, although there are many related articles in the journal of *IEEE Access*, its average times cited is only 14.9, indicating that the quality and influence of these articles are generally not too high.

**Table 3:** The top 10 journals with literature quantity

	Name of Journal	Literature quantity	GCS	Average times cited
1	*Sensors*	424	8391	19.8
2	*IEEE Access*	237	3531	14.9
3	*IEEE Internet of Things Journal*	112	2792	24.9
4	*IEEE Sensors Journal*	110	2596	23.6
5	*Future Generation Computer Systems-the International Journal of Escience*	85	1812	21.3
6	*JMIR Mhealth and Uhealth*	77	679	8.8
7	*IEEE Journal of Biomedical and Health Informatics*	66	2089	31.7
8	*Journal of Medical Systems*	65	885	13.6
9	*Plos One*	52	1504	28.9
10	*Acs Applied Materials & Interfaces*	48	1237	25.8

Overall, IEEE is the biggest winner in the field of smart health research based on IoT. In addition, there are many journals that focus on the IoT, sensors and health, but there are few journals focusing on the intersection of the IoT and health. This shows that the research on the field of smart health research based on IoT has not yet had a great influence, and the major journals have not paid much attention to it.

### Knowledge base analysis

Co-citation Networks refers to a knowledge network formed by two scientific documents simultaneously cited by the third article [29]. Literature co-citation analysis expresses the relationship between documents by the frequency cited by other literatures. That is to say, a certain two documents are cited together by several other documents. The higher the frequency of citations, the closer the relationship between the two documents is, which means that the more the subject backgrounds of the two documents are similar [30]. Fundamentally speaking, when certain documents, journals, academic groups or individuals are repeatedly quoted by their peers, the knowledge carriers that are cited are essentially recognized by the scientific community in which they are located, thus forming a scientific paradigm. This paradigm relationship can be visualized by analysis of the co-citation network of the literature [31]. Therefore, through the literature co-citation network analysis, the knowledge base of the research on the field of smart health research based on IoT can be concretely demonstrated.

**Fig. 6:**
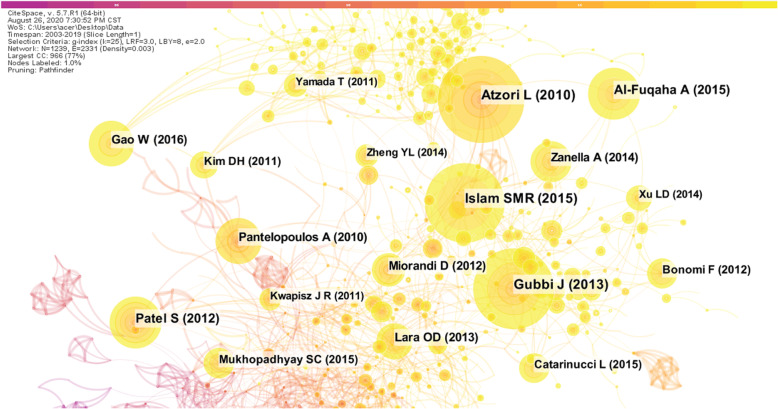
Articles in the co-citation network

We import the preprocessed data into CiteSpace, analyze the co-citation relationship between scientific literatures, and generate a co-citation network diagram of the literature, as shown in Fig. 6. In the figure, each node represents a document that is commonly cited. The size of the node is proportional to the number of times it is cited, the connection between the nodes indicates a co-citation relationship. The thickness of the connection indicates the strength of the co-cited, and the different colors indicate the year in which the document was cited. The number of network nodes is 1239, the number of connections between nodes is 2331, and the density of the network is 0.003.

Table 4 lists the top 10 co-citation literature and related information. In the literature citation network, Atzori’s article published in *Computer Networks* in 2010 titled “The internet of things: A survey” was cited as the highest frequency, reaching 290 times [1]. So far, this article has been cited as a total of 13,691 times in Google Scholar. Gubbi [32], Islam [33], Patel [34], Al-Fuqaha [35], etc., which are ranked behind, are connected to Atzori [1]. It shows that the correlation between the top citations in the field is very strong, and the topics of the scientific literature are similar. These documents are all about IoT technology and applications. It can be seen that the current research on IoT technology has been initially matured and standardized. Interestingly, five of the top 10 cited articles were published by the IEEE Publishing Group.

**Table 4:** List of the top 10 co-citation articles with the corresponding frequencies

Author	Year	Name of Journal	Frequency
Atzori L	2010	*Computer Networks*	290
Gubbi J	2013	*Future Generation Computer Systems-The International Journal of eScience*	269
Islam SMR	2015	*IEEE Access*	262
Patel S	2012	*Journal of NeuroEngineering and Rehabilitation*	177
Al-Fuqaha A	2015	*IEEE Communications Surveys and Tutorials*	171
Pantelopoulos A	2010	*IEEE Transactions on Systems Man and Cybernetics Part C-applications and Reviews*	159
Gao W	2016	*Nature*	150
Zanella A	2014	*IEEE Internet of Things Journal*	138
Lara OD	2013	*IEEE Communications Surveys and Tutorials*	126
Miorandi D	2012	Ad Hoc *Networks*	115

Combined with the above-mentioned journal analysis, it can be said that the IEEE Publishing Group has made tremendous contributions to the development of this field. In addition, in terms of centrality, Atzori’s performance is also very good, the greater the centrality of a node in the network, indicating that it is more important in the network [36]. Therefore, the comprehensiveness of all aspects can reflect the importance of the document Atzori [1]. It can be said that this article lays the foundation for the research of smart health research based on IoT. In general, the literature of Fig. 6 has a relatively tightly distributed network, indicating that the knowledge base in this field has been initially formed, which will provide important knowledge support for subsequent research.

### Research focus analysis

Research hotspots refer to the focus of research in a certain discipline in a certain period of time. Generally speaking, there is a large number of scientific literatures, academic thoughts, and research groups emerging on a subject [37]. Kuhn [38] emphasized that the development of science is an alternating appearance of the conventional science and the scientific revolution, which indicates that the scientific revolution is changing, and that the old and new paradigms are incommensurable. It is precisely because of the existence of incommensurability that the vocabulary system between the old and new paradigms will change accordingly. So, whether the scientific revolution occurs can be judged from whether the vocabulary of the period has changed. The number of co-occurrences of different keywords in the scientific literature can be counted. The level of the co-occurrence frequency can reflect the correlation between the keywords and the hot issues in the specific field during this period [39]. Therefore, the co-occurrence analysis of keywords can reveal the research structure and research hotspots in specific fields. The research results of Callon et al. [40] are the earliest applications of co-word analysis. Subsequently, the co-word analysis method has been widely used in the field of information science. Of course, keyword analysis is also based on certain assumptions [41], including: (1) The selection of keywords is cautious; (2) Multiple keywords in the same document are related to each other and are recognized by the author; (3) If enough authors recognize the relationship between the same keyword, then this relationship can be considered to have a certain meaning in the field; (4) The keyword can reflect the content of the document to a certain extent. When the author chooses the keyword, it is usually affected by other research results. The basic principle of co-word analysis is to count the number of times a group of keywords appears in the same group of documents, then the degree of co-occurrence is measured by the number of co-occurrences, the more co-occurrences, the more closely they are related [42].

The key word is a high degree of conciseness and generalization of an article, which is the core and essence of the article, frequently high keywords are often used to identify hot topics in a research field. By analyzing keywords, we can intuitively grasp the main research content of a paper, and even the overall research situation in a field [43]. This study extracts the key words from 9561 documents and conducts frequency statistics and frequency co-occurrence analysis to understand the current structural foundations and research hotspots in the field of smart health research based on IoT, and predict the future development direction of the field. Table 5 lists the top 20 keywords of the co-occurrence frequency. It can be seen that the keyword with the highest frequency is the internet of things, this is very consistent with the topic of this article. The subject of this paper is the IoT and health. The IoT is also the keyword with the highest centrality, indicating that research in this field is basically carried out around the IoT. These keywords with high frequency of occurrence can be divided into four main categories: (1) Keywords related to the IoT technology, such as internet of thing, sensor, wireless sensor network, big data, cloud computing, etc.; (2) Keywords related to health, such as healthcare, health, parkinsons disease, etc.; (3) Keywords related to smart health, such as smart home; (4) Problems arising from research in the field of smart health research based on IoT, such as security.

**Table 5:** List of the top 20 keywords with the corresponding frequency

	Keyword	Frequency		Keyword	Frequency
1	internet of thing	2329	11	technology	351
2	wearable sensor	812	12	security	345
3	system	715	13	accelerometer	313
4	healthcare	670	14	wearable	290
5	sensor	644	15	machine learning	272
6	internet	580	16	wireless sensor network	271
7	wearable device	563	17	cloud computing	268
8	health	430	18	big data	264
9	physical activity	366	19	smart home	263
10	thing	352	20	parkinsons disease	256

**Fig. 7:**
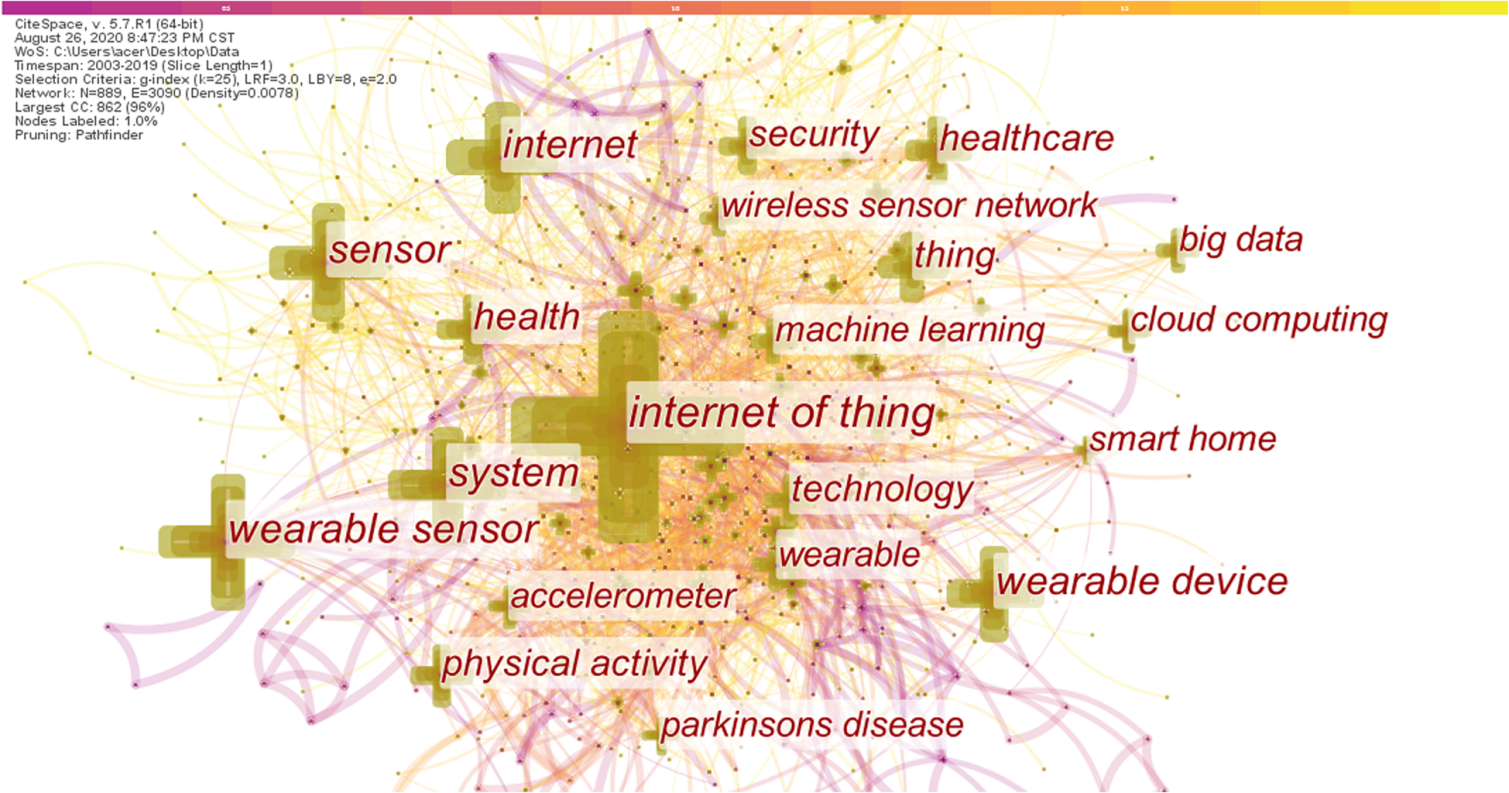
Keyword co-occurrence network

The co-word network refers to an objective knowledge network that expresses the structure of the scientific knowledge domain, which is composed of co-occurrence between keywords. It can be used to describe the knowledge structure of a subject domain and can reveal the evolution of a disciplinary structure in combination with time series [44]. We import the pre-processed data into CiteSpace to analyze the keywords of the scientific literature and generate a keyword co-occurrence network diagram, as shown in Fig. 7. Each node represents a different keyword, and the size of the node is proportional to the frequency of its co-occurrence. The connection between nodes indicates the co-occurrence relationship between two keywords in the same document, and the different colors indicate the years in which different keywords co-occur. The number of network nodes is 889, the number of connections between nodes is 3090, and the density of the network is 0.0078. It can be seen from the figure that the network as a whole is relatively dense, and the co-occurrence relationship between multiple groups of keywords is relatively close, indicating that the research results in the field of intelligent health research based on the Internet of Things are mostly multi-theme research.

In the future research, the IoT is still the center of research in this field. Focusing on the integration of the IoT and the integration of smart health, it is necessary to solve the security and privacy issues in this field and eliminate concerns for users. IoT technologies often appear at the same time as cloud computing, big data, etc. These emerging technologies are closely related, and artificial intelligence technology cannot lack them. Therefore, in future research, the IoT, big data and cloud computing will frequently appear in the research in this field. Smart homes and smart cities have received a lot of attention in recent years. Smart home is a platform for housing, using IoT technology to connect various devices in the home to achieve convenience, comfort and intelligence. At present, there are few studies on the use of IoT technology to treat specific diseases (such as parkinsons diseases appearing in the top 20 keywords, etc.), so this may be one of the future research trends.

## Discussions

### Summary of findings

Using HistCite, CiteSpace, Excel and other analysis tools, the time distribution, spatial distribution, literature citation and research hotspots of knowledge in this field are deeply analyzed and visualized.

(1) In terms of time distribution: the annual load capacity curve and the annual author input curve change trend are roughly the same, and the overall growth trend. In particular, the growth rate since 2014 has been very rapid, almost showing a linear upward trend, much higher than the index trend line. Explain that research in this field will continue to increase in the future and that it will remain a hotspot for future research.

(2) In terms of spatial distribution: a) Author distribution: The author’s cooperation network is sparse, the cooperation between the authors is not close enough, and the stable core author group has not yet formed in this field; b) Institutional distribution: Similarly, the cooperation and cooperation between institutions is not close enough, and a stable and mature institutional cooperation relationship has not yet been formed; c) Journal distribution: There are few journals focusing on the intersection of IoT technology and health. This shows that the research in this field has not yet had a great influence, and the major journals have not paid much attention to it.

(3) In terms of knowledge base analysis: the literature has a relatively tightly indexed network, and the knowledge base in this field has been initially formed, which can provide important knowledge support for subsequent research.

(4) In terms of research hotspot analysis: high-frequency keywords can be divided into four parts. The Internet of Things is a keyword with the highest co-occurrence frequency and the highest centrality. In addition, most of the research results in this field are multi-theme research. Among them, smart home and the use of IoT technology to assist in the treatment of specific diseases are the future research trends.

### Future trends

Through the research in this paper, we can find that smart home and smart city are the research hotspots in recent years, and will likely also be the focus of future research.

Smart homes can be a “family health consultant”. For example, the smart home system can realize the “alarm” of the elderly and children, notify the family and locate; The system will automatically start and shut down the air purifier according to the real-time air condition, without manual operation; In addition, the air purifier can be controlled based on the humidity level in the house and the PSI level of asthma and allergic rhinitis patients [45]. The smart screen in the kitchen can see the children in the living room through the security system, and is equipped with detection equipment for harmful gases such as gas to achieve the function of safety protection; The smart clothing care machine in the cloakroom has the functions of steam sterilization, shaping, drying and other functions to protect the health of users.

The construction of smart health protection system is an important part of the construction of smart cities. The construction of “digital health” system is the focus of promotion. The smart city will build a medical health big data platform, realize the data sharing and sharing of medical and health service organizations, and rely on the smart city cloud platform to form a citizen medical health information big data center, and provide support for promoting three-medicine linkage and achieving graded diagnosis and treatment. In addition, electronic health records of residents in the city will be established to realize the networking of health services in hospitals and clinics throughout the city. And promote remote registration, electronic toll collection, online telemedicine services, graphic and physical examination diagnostic systems, etc., to comprehensively improve the city’s medical and health services.

### Future works

The following work will be done in the future studies: (1) The first one it to examine how the Internet of Things actually affects medical accessibility; (2) The second one is to evaluate the objectiveness or reproducibility of reported results as well as relationship between prominent authors and industry; (3) The third one is to use more data resources rather than only WoS data and further validate our search result; (4) The fourth but not the last one is to eliminate irrelevant search results with technical tools rather than the manual methods used on this study.

## Conclusions

In order to explore the knowledge base, research hotspots, development status and future research directions of the research on the field of smart health research based on IoT. We conducted a bibliometric analysis of 9561 articles in the Web of Science core database for the 17 years from 2003 to 2019. The results show that the field of smart health research based on IoT has developed rapidly since 2014, but has not yet formed a stable network of authors and institutions. Our research provides panoramic knowledge support for researchers in related fields to understand the research status, future trends and hotspots in the field of smart health research based on IoT.

## Data Availability

Data materials are available from the lead author upon request.

## Abbreviations

IoT: Internet of things
MAS: Multi-agent system
COPD: Chronic obstructive pulmonary disease
CoSHE: Cloud-based smart home environment
IIoT: Industrial Internet of Things
WoS: Web of Science
LCS: Local citation score
GCS: Global citation score
